# Sources of Stigma and its Relation to Internalized Stigma in Women with Borderline Personality Disorder

**DOI:** 10.62641/aep.v53i4.1926

**Published:** 2025-08-05

**Authors:** Elia Solís-Villegas, María Yoldi-Negrete, Iván Arango de Montis, Ilyamín Merlín-García, Carlos-Alfonso Tovilla-Zárate, Ana Fresán

**Affiliations:** ^1^Dirección de Enseñanza, Instituto Nacional de Psiquiatría Ramón de la Fuente Muñiz, 14370 Mexico City, Mexico; ^2^Subdirección de Investigaciones Clínicas, Instituto Nacional de Psiquiatría Ramón de la Fuente Muñiz, 14370 Mexico City, Mexico; ^3^Dirección de Servicios Clínicos Instituto Nacional de Psiquiatría Ramón de la Fuente Muñiz, 14370 Mexico City, Mexico; ^4^División Académica Multidisciplinaria de Comalcalco, Universidad Juárez Autónoma de Tabasco, 36040 Comalcalco, Tabasco, México

**Keywords:** borderline personality disorder, stigma, internalized stigma, demographics

## Abstract

**Background:**

Borderline personality disorder (BPD) has a high prevalence, presenting with self-injurious behaviors, suicide attempts and other psychiatric comorbidities. This condition is accompanied by high levels of stigma and self-stigma, driving to deleterious effects on prognosis. The present study aimed to compare demographic and clinical characteristics of women diagnosed with BPD with low and high internalized stigma and to address internalized stigma dimensions severity (disclosure, positive aspects, and discrimination) between the source of perceived stigma.

**Methods:**

A total of 106 women with a diagnosis of BPD were evaluated for sociodemographic data, sources of stigma, the severity of the symptomatology and internalized stigma, evaluated with the Borderline Evaluation of Severity Over Time (BEST) and the Spanish version of King's Internalized Stigma Scale (ISS), respectively.

**Results:**

Participants with high internalized stigma reported greater symptom severity. Regarding the reported sources of stigma, in almost all sources of stigma, discrimination was perceived as greater (*p* < 0.05) (friends, co-workers, doctors, psychiatrists and nurses) as well as the perceived global internalized stigma (*p* < 0.05) for friends, co-workers and nursing staff.

**Conclusion:**

It is necessary to address stigmatizing behaviors by health personnel and the support network close to the patient in addition to improving awareness about associated internalized stigma which is related to worse outcomes.

## Introduction

Borderline personality disorder (BPD), the most diagnosed personality disorder, is present in approximately 1.6% of the population, and is more common in women [1]. Approximately 30% of patients present self-harm behaviors [2, 3] and risk of suicide remains as high as 10% over a 27-year course [2, 4] with a prevalence of suicidality of 75% [5]. In addition, comorbidities such as depressive episodes, anxiety disorders and substance use disorders are the rule rather than the exception, making this condition especially complex [6, 7, 8].

Furthermore, BPD has been described as the most stigmatized psychiatric diagnosis: in studies comparing internalized stigma in patients with schizophrenia, depressive or anxiety disorders and BPD, the latter shows higher levels of internalized stigma globally, in addition to greater symptom severity compared to the other groups [9, 10]. Quenneville *et al*. [11] compared internalized stigma in patients with BPD, attention deficit hyperactivity disorder and bipolar disorder. Patients with BPD presented with higher internalized stigma, especially in those areas consisting of perceived discrimination, social isolation, and resistance to stigma.

Stigma can come from various sources; patients with BPD frequently experience stigmatizing behaviors from family members and friends, such as loss of autonomy, overprotective behavior, or physical and verbal abuse. In addition, some studies show that family members’ own stress regarding BPD symptoms may lead them to isolate themselves from the patient, thus contributing to perpetuating stigmatizing behaviors [12, 13]. The medical population has been signaled as an important source of stigma: medical personnel such as psychiatrists, psychologists, nurses, and other specialists, have been found to have a negative perception of patients with this diagnosis [14, 15, 16]. Finally, it is known that even in the workplace, patients may suffer from discrimination from employers and coworkers, making it more difficult to get or keep jobs and preferring to hide the diagnosis most of the time [17, 18]. Living in a society with stigmatizing ideas towards BPD, people with this diagnosis tend to internalize stigma. Internalized stigma is characterized by negative feelings about oneself, maladaptive behaviors and application of negative stereotypes because of individual experiences or anticipation of a negative social reaction, in this case, about mental illness [19, 20]. Internalized stigma is associated with increased emotional instability, decreased self-esteem, hopelessness and diminished social adaptation and quality of life [21]. In summary, internalized stigma greatly changes the illness course, attained functioning and overall prognosis of individuals affected with BPD.

As internalized stigma is passively acquired and depends on the living experience, its core characteristics and severity might be influenced by the perceived source of stigmatization [19]. However, to the best of our knowledge, no studies have addressed this topic.

Therefore, the aims of the present study were: (1) to compare demographic and clinical characteristics of women diagnosed with BPD with low and high internalized stigma and, (2) to address internalized stigma dimensions severity (disclosure, positive aspects, and discrimination) between the source of perceived stigma.

Our hypotheses were that participants with higher internalized stigma would report a higher number of psychiatric hospitalizations and more severe symptomatology related to the personality disorder and that participants would report higher stigma from persons not related to the healthcare system, including relatives, friends and coworkers/schoolmates. Finally, that *discrimination* would be higher if the stigmatizing behavior was perceived to come from healthcare personnel and *disclosure* would be higher among those who perceived stigma from sources as family members and friends.

## Methods

### Study Design and Participants

For the present cross-sectional study, a total of 106 women over 18 years, with a diagnosis of BPD according to DSM-5 criteria [22], confirmed by the medical chart and treating psychiatrist were recruited from the Borderline Personality Clinic an outpatient service at the *Ramón de la Fuente Muñiz* National Institute of Psychiatry (INPRFM). Women were not included if they had, in accordance with the clinical chart and last clinical note, psychotic symptoms, bipolar disorder, manic symptoms, or were agitated at the time of the evaluation for the study. We exclude these clinical features to avoid biases in the assessment of internalized stigma, which was expected to be answered according to their experiences with a diagnosis of BPD.

Participants were recruited using a non-probabilistic sample approach. They took part voluntarily after a full explanation of the aim and procedures of the study. After their verbal acceptance, they all gave written informed consent to participate. The study was approved by the INPRFM Research Ethics Board (CONBIOÉTICA-09-CEI-010-20230316) following the ethical principles and guidelines of the Declaration of Helsinki.

### Assessment Procedures

Diagnosis of BPD was undertaken in the Borderline Personality Clinic according to DSM-5 criteria, confirmed by the medical chart and treating psychiatrist. Demographics, clinical variables, and sources of stigma analyzed in this study were obtained through a clinical face-to-face interview with the participant and her relatives. Questions related to demographic status included current age, years of education, marital and employment status; while clinical variables assessed through a clinical interview were linked to clinical features during illness evolution also assessed in the BPD Clinic, including psychiatric comorbidity (depressive and anxiety disorders, previous suicide attempts, self-injurious behaviors, previous psychiatric hospitalizations, age at first hospitalization and history of sexual abuse).

Sources of stigma were assessed with an ad hoc descriptive instrument developed for the present study. It included family members, friends, co-workers/schoolmates, psychiatrists, other physicians, and psychiatric nursing staff as sources of stigma. A visual analog scale, from 1 (the least stigma perceived) to 10 (the most extreme stigma perceived), was used to assess the level of stigma perceived from each of the previous sources of stigma described.

Symptom severity at the time of the study was assessed with the Borderline Evaluation of Severity Over Time (BEST) [23]. It is a 15-item self-report instrument scored on a five-point Likert scale designed to measure three subscales: (A) Thoughts and Feelings (first eight items rated from 1 = None/Slight to 5 = Extreme), including mood reactivity, identity disturbance, suicidal thoughts, unstable relationships and emptiness; (B) Behaviors-Negative (next four items rated from 1 = None/Slight to 5 = Extreme), evaluating self-harm behaviors; and (C) Positive Behaviors (final three items rated from 5 = Almost Always to 1 = Almost Never). The BEST has shown adequate reliability for its use in Mexican patients with BPD [24]. The internal consistency of the BEST subscales for the present study ranged from an alpha of 0.75 to 0.85.

For the assessment of internalized stigma, the Spanish version of King’s Internalized Stigma Scale (ISS) was used [25, 26]. It comprises 28 self-report items of the ISS scored on a five-point Likert agreement scale that assess three dimensions: (1) Discrimination (13 items) *which refers to the perception of negative reactions of other people toward the illness, including acts of discrimination by health professionals and employers*, (2) Disclosure (10 items) *includes questions regarding embarrassment or feeling bad about the illness and managing disclosure to avoid discrimination*, and, (3) Positive aspects (5 items) *which refers to how the person accepts the illness and perceive himself/herself as less affected by stigma* [25]. The total score is obtained by the sum of the item scores (some of them reverse-coded) with higher scores reflecting higher internalized stigma. The internal consistency of the ISS dimensions and total score for the present study ranged from an alpha of 0.74 to 0.81. Until now, there is no cut-off point of the total score to define levels of stigma. Therefore, we decided to use the median score of the scale to divide the sample into those women with high internalized stigma and those with low internalized stigma. Median splits do tend to give the best results as a cutoff point when the original variable has a symmetric distribution [27]. The Kolmogorov-Smirnov test showed a normal distribution of the total score of the ISS in this sample (*p* = 0.20). Although this is an artificial categorization of a continuous variable, the cost in terms of power is relatively small and justified by the improvements it offers in interpretability [27].

### Statistical Analysis

Descriptive values of all variables were determined with frequencies and percentages for categorical variables and means and standard deviations (S.D.) for continuous variables. For the comparison between low and high internalized stigma groups, chi-square tests (χ^2^) followed by Fisher’s exact test and Mann-Whitney U tests or independent-sample *t*-tests were used where appropriate. These analyses were also used to compare the dimensions and the total score of the ISS according to the identified sources of stigma (not perceived stigmatization vs. perceived stigmatization). Mann Whitney U tests were used for the comparison of number of suicide attempt, number of psychiatric hospitalizations and age at first psychiatric hospitalization as the Kolmogorov-Sminov tests exhibited non-normal distributions for these variables (*p* < 0.05). All analyses were deemed significant with a *p*-value ≤ 0.05 and were performed with the IBM SPSS Statistics for Windows, Version 25.0. Armonk, NY, USA: IBM Corp.

### Patient and Public Involvement

Women with BPD were involved in the research with the interpretation of the data. After the assessment performed for the research, some of the women included in the sample, gave information about their experiences of discrimination with the main sources of stigma. This information, not included for a qualitative analysis, was useful in giving meaning to the interpretation of the data beyond what was described in previous scientific articles.

## Results

### Demographic and Clinical Features Among Women With BPD With Low or High Internalized Stigma

A total of 106 women diagnosed with BPD were included in the study. The mean age of the sample was 30.5 (S.D. = 10.4) years with 14.2 (S.D. = 3.2) years of education. Most of the participants were single at the time of the study (84.0%, *n* = 89) and without an economically remunerated activity (66.0%, *n* = 70).

Most of the participants reported psychiatric comorbidities, particularly depressive and anxiety disorders. Of the included participants, 81.1% (*n* = 86) had a history of at least one previous suicide attempt, and 90.6% (*n* = 96) reported self-harm behaviors. Just over half of the participants (54.7%, *n* = 58) had had a previous psychiatric hospitalization, with the first hospitalization at age of 24.6 (S.D. = 8.0), and had a history of sexual abuse (54.7%, *n* = 58).

The mean scores of the ISS dimensions were as follows: Discrimination (30.6, S.D. = 8.9), Disclosure (24.3, S.D. = 7.1), Positive aspects (8.4, S.D. = 3.7), and the total score of the ISS (63.4, S.D. = 14.0). The median score of the ISS was 63, which was used to divide the sample into women with low internalized stigma and women with high internalized stigma.

The comparison of demographic and clinical features, including current symptom severity assessed with the BEST scale, between these groups are shown in Table 1. Both groups display similar demographic and clinical features. Women with high internalized stigma exhibited higher symptom severity in the dimensions: thoughts and feelings, behaviors-negative and the total BEST score. No differences arose in the dimension of behaviors-positive (see Table 1).

**Table 1.** **Demographic and clinical features between women with low and high internalized stigma**.

		Low Stigma		High Stigma		Statistics
		n = 51		n = 55		
Demographic features						
Age (years) *mean, S.D.*		29.6	9.1	31.4	11.4	t = –0.8, *p* = 0.30
Years of education (years) *mean, S.D.*		14.6	3.7	13.8	2.7	t = 1.1, *p* = 0.23
Marital status – single *n %*		41	80.4	48	87.3	χ^2^ = 0.9, *p* = 0.33
						*Fisher’s *p* = 0.42
Employment status – not remunerated *n %*		32	62.7	38	69.1	χ^2^ =0.4, *p* = 0.49
						*Fisher’s *p* = 0.54
Clinical features						
Psychiatric comorbidities (yes – *n* = 105)		50	98.0	55	100	χ^2^ = 1.0, *p* = 0.29
						*Fisher’s *p* = 0.48
	Depressive disorder – yes *n %*	49	98.0	53	96.4	χ^2^ = 0.2, *p* = 0.61
						*Fisher’s *p* = 1.00
	Anxiety disorder – yes *n %*	26	52.0	24	43.6	χ^2^ = 0.7, *p* = 0.39
						*Fisher’s *p* = 0.43
Previous suicide attempt – yes *n %*		41	80.4	45	81.8	χ^2^ = 0.03, *p* = 0.85
						*Fisher’s *p* = 1.00
Number of suicide attempts *mean, S.D.*		3.2	2.7	3.3	2.4	^+^U = 876, *p* = 0.68
Self-harm behaviors – yes *n %*		46	90.2	50	90.9	χ^2^ = 0.01, *p* = 0.90
						*Fisher’s *p* = 1.00
Psychiatric hospitalization – yes *n %*		25	49.0	33	60.0	χ^2^ = 1.2, *p* = 0.25
						*Fisher’s *p* = 0.32
Number of hospitalizations *mean, S.D.*		1.4	0.8	2	1.5	^+^U = 303.5, *p* = 0.06
Age at 1st hospitalization *mean, S.D.*		24.4	6.7	24.8	9.0	^+^U = 395.5, *p* = 0.78
History of sexual abuse – yes *n %*		25	49.0	33	60.0	χ^2^ = 1.2, *p* = 0.25
						*Fisher’s *p* = 0.32
Symptom severity (BEST scale, *mean, S.D*.)						
Thoughts and Feelings		26.4	7.2	30.2	6.2	t = –2.8, *p * < 0.01
Behaviors – Negative		10.5	4.9	13.1	4.7	t = –2.7, *p * < 0.01
Behaviors – Positive		10.4	2.6	9.9	2.3	t = 0.9, *p* = 0.34
Total score		41.1	11.6	48.6	10.9	t = –3.4, *p * < 0.01

* Fisher’s exact test *p*-values. ^+^ Mann-Whitney U-tests.

### Internalized Stigma According to the Main Sources of Stigma Identified by Women With BPD

The main source of stigma reported were: family members (92.5%, *n* = 98; mean level of stigma 7.4, S.D. = 2.4), followed by friends (81.1%, *n* = 86; mean level of stigma 6.6, S.D. = 2.3), co-workers/schoolmates (75.5%, *n* = 80; mean level of stigma 7.5, S.D. = 2.2), other physicians (57.5%, *n* = 61; mean level of stigma 5.8, S.D. = 2.7), psychiatrists (46.2%, *n* = 49; mean level of stigma 4.6, S.D. = 2.6) and nursing staff (45.3%, *n* = 48; mean level of stigma 5.2, S.D. = 2.7).

ISS dimensions and total scores were compared between women who perceived stigmatization from either one of the previous sources and those who did not. Perceived stigma from either friends, other physicians, or nursing staff, was associated with higher internalized stigma scores. Regarding the ISS dimensions, discrimination was higher among those who perceived being stigmatized by all sources except for family members. Disclosure was higher among those who perceived stigma from family members and friends, Positive Aspects were lower in women who perceived stigma from other physicians The comparison between groups is shown in Table 2.

**Table 2.** **Internalized stigma according to the source of perceived stigmatization**.

Internalized Stigma Scale	Not Perceived Stigmatization		Perceived Stigmatization		Statistic
Family members (*mean, S.D.*)					
Discrimination	26.2	8.1	31.0	8.9	t = –1.4, *p* = 0.14
Disclosure	19.5	7.0	24.7	7.0	t = –2.2, *p* = 0.04
Positive aspects	8.7	4.1	8.4	3.7	t = 0.2, *p* = 0.80
Total score	54.5	13.0	64.1	13.9	t = –1.9, *p* = 0.06
Friends (*mean, S.D.*)					
Discrimination	23.8	8.1	32.2	8.3	t = –4.0, *p * < 0.01
Disclosure	20.1	5.8	25.3	7.1	t = –3.0, *p * < 0.01
Positive aspects	9.7	4.3	8.1	3.6	t = 1.7, *p* = 0.08
Total score	53.7	12.2	65.7	13.4	t = –3.6, *p * < 0.01
Co-workers/Schoolmates (*mean, S.D.*)					
Discrimination	27.4	7.7	31.7	9.1	t = –2.1, *p* = 0.03
Disclosure	23.6	5.9	24.5	7.5	t = –0.5, *p* = 0.58
Positive aspects	8.9	4.2	8.2	3.6	t = 0.7, *p* = 0.45
Total score	60.0	12.1	64.5	14.4	t = –1.4, *p* = 0.15
Other physicians (*mean, S.D.*)					
Discrimination	26.6	8.2	33.6	8.2	t = –4.2, *p * < 0.01
Disclosure	24.2	7.1	24.4	7.2	t = –0.1, *p* = 0.84
Positive aspects	9.3	4.0	7.7	3.4	t = 2.1, *p* = 0.03
Total score	60.2	13.8	65.8	13.7	t = –2.0, *p* = 0.04
Psychiatrists (*mean, S.D.*)					
Discrimination	28.1	7.5	33.6	9.5	t = –3.3, *p * < 0.01
Disclosure	24.5	7.3	24.1	7.0	t = 0.2, *p* = 0.77
Positive aspects	8.6	3.8	8.2	3.7	t = 0.4, *p* = 0.63
Total score	61.2	13.2	66.0	14.5	t = –1.7, *p* = 0.08
Nursing staff (*mean, S.D.*)					
Discrimination	28.6	8.4	33.1	8.9	t = –2.6, *p* = 0.01
Disclosure	23.5	7.5	25.3	6.6	t = –1.2, *p* = 0.21
Positive aspects	8.7	4.0	8.0	3.4	t = 0.9, *p* = 0.36
Total score	60.9	14.5	66.4	12.8	t = –2.0, *p* = 0.04

## Discussion

In this study we sought to compare demographic and clinical characteristics of women diagnosed with BPD with low and high internalized stigma and, to address internalized stigma dimensions severity (disclosure, positive aspects, and discrimination) between the source of perceived stigma.

Our findings support that women with BPD with higher internalized stigma exhibit greater symptom severity in the dimensions of thoughts and feelings and negative behaviors of the BEST scale. These symptoms dimensions are taken from the DSM and even though the mean scores reflect moderate symptom severity, women with high internalized stigma may exhibit more pronounced mood reactivity, behaviors associated to unstable relationships and other negative behaviors which may result in impaired functioning. These symptoms can increase internalized stigma, as they can involve attitudes and behaviors in others that the person with BPD may perceive as rejection [10, 11, 18].

Being a process in which the person develops concepts about mental illness from an early age, this largely as part of socialization, expectations begin to be internalized or to anticipate that an individual with mental illness is rejected in society, as a friend, employee or as a member of society in general, making this devaluation later personal and relevant when developing a mental illness and perceiving such concepts from outsiders, regardless of the stigmatizing source [20].

According to our study it seems that internalized stigma is more severe if the person perceives herself as being stigmatized by externals, especially from a close support network such as family members. Despite the above, the clinical complexity of the personality condition and its associated behaviors often result in the family experiencing elevated levels of negative expressed emotion, frustration, and a heightened perceived burden among family members. This added strain can intensify the emotional and psychological challenges faced by those close to individuals with BPD. The disorder’s emotional intensity and unpredictability can make it difficult to maintain supportive relationships, leaving relatives feeling overwhelmed. This, in turn, can worsen the stigma both the patient and their family experience [28]. Most family members may blame themselves or isolate themselves from the patient diagnosed with BPD, which derived from the above, could unconsciously lead to negative and self-stigmatizing behaviors in patients [29, 30]. In this study the second source reported were friends. Some studies reported that discrimination is the most commonly experienced in interpersonal relationships, with 33–50% reported by friends [30]. The most common perceived by patients with a psychiatric disorder is social distancing, varying from a reduction in social contact or exclusion from certain events. In this way, higher scores on the Disclosure dimension may reflect the effort of patients with BPD in managing information about their diagnosis to avoid social discrimination from their friend and to avoid the perpetuation of negative stereotypes in relation to the mental illness [29]. It may be that by having a greater affective bond with friends (which may even be part of the patient’s support network) a greater perception of rejection is perceived, increasing the levels of internalized stigma compared to the other sources studied. In line with the above, Positive Aspects was higher in participants with perceived support from family, friends or employers.

Coworkers were reported as the third source of stigma: it has been reported that a significant number of employers perceive individuals with psychiatric disorders as incompetent, incapable of coping, and unaccountable, or requiring constant supervision [17]. It is possible that, being a professional relationship, there is less tolerance for the exacerbation of symptoms or negative behaviors carried out by patients, which in turn can increase the perception of rejection in patients [18]. Finally, other physicians were reported as a source of stigma. Throughout the literature, it has been reported that the symptoms and behaviors inherent to BPD cause strong emotional reactions in hospital staff, in addition to being seen as exclusively psychiatric patients and often as manipulative [31, 32, 33]. In addition to the above, there is a lack of empathy and understanding on the part of health personnel towards self-injurious behaviors, characteristics of these patients: discriminatory behaviors and longer waiting times by health personnel have been reported, especially in emergency environments, when the patient present with self-harm [34, 35]. It seems then that only highly trained personnel such as psychiatric nurses or psychiatrists would be able to avoid stigmatizing behaviors. This could be related to the lack of information prevailing in the general and health personnel population regarding the disorder and it can contribute to the expressions of these sources of increasing perceived stigma. There is often the belief that people with BPD are dangerous to themselves and to others and the idea that they are difficult patients to work with, who manipulate and seek attention [10, 14, 36]. The above suggests that the difficulty in interacting with patients with this diagnosis stems largely from the stigma related to the diagnosis [21, 37]. The interactions with these patients frequently generate negative countertransference responses, which, added to the lack of training and specific resources for patients with this disorder, could generate negative expressions towards these patients [38].

Interestingly, the dimensions of internalized stigma may vary depending on the stigmatizing source: the aspect of discrimination was higher among those who perceived being stigmatized by most sources except for family members. We hypothesize that this could be related to the greater affective bond: it is possible that family members have a greater tolerance of negative behaviors carried out by patients, which could lead to a lower perception of rejection or that patients present greater habituation to stigmatizing comments or behaviors from family members compared to third parties. Disclosure of a psychiatric diagnosis, for instance, BPD, tends to be higher among individuals whose sources of stigma come from family members and friends. This is particularly notable because, in some studies, disclosing a psychiatric condition to close social circles can have unintended negative consequences. The act of sharing such personal information may lead to rejection, as family members or friends may harbor misconceptions or prejudices about mental health conditions. This rejection can, in turn, lead to poor social support, further isolating the individual at a time when emotional and practical support is most needed [39].

Internalized stigma in patients with BPD can significantly affect their emotional well-being, treatment engagement, and social relationships. It often amplifies feelings of shame, guilt, and worthlessness, which are already common in individuals with BPD [21]. This emotional distress can worsen symptoms of depression, anxiety, and emotional dysregulation, making it harder for patients to manage their condition effectively [40]. As a result, these patients may be less inclined to seek help or may prematurely discontinue treatment, believing their condition is too shameful or that they are beyond help [41]. Moreover, reluctance to engage in treatment can undermine the effectiveness of therapeutic interventions, as recovery requires active participation and trust in the process [42].

Addressing internalized stigma in patients with BPD is essential for improving treatment outcomes. Therapeutic approaches such as dialectical behavior therapy (DBT) [42] or schema therapy [43] can assist individuals in challenging and reframing negative beliefs about themselves and their disorder. These therapies foster emotional regulation, self-acceptance, and the development of healthier coping mechanisms [42, 43, 44].

Psychoeducation plays a crucial role in helping patients cope with internalized stigma by normalizing their experiences and reframing the disorder as something that can be managed [44]. Educating patients about BPD, its causes, and its treatability can reduce feelings of shame and empower them in their recovery. Additionally, educating those close to patients—such as family members, friends, schoolmates, and coworkers—can enhance understanding, reduce negative attitudes, and promote a more supportive and empathetic environment [44]. This, in turn, can help counteract the harmful effects of both social and internalized stigma.

Concerning limitations, our results may not be generalizable to all women with BPD. First, the sample size is limited, and we excluded a proportion of patients whose clinical presentations may have been more complex at the time of the study (e.g., psychotic symptoms). Additionally, recruitment was conducted at a single tertiary-level mental health center, and the cross-sectional design of the study does not allow for the identification of causal factors contributing to internalized stigma.

We must state the fact that stigmatizing sources were perceived as such by patients, with no formal evaluation of the attitudes and conceptions towards BPD of those involved, which could ascertain or disprove the perception of patients. It is possible that the hypersensitivity inherent to the condition may cause them to perceive discrimination where others would not. Furthermore, discrimination from others might come from a history of past encounters with unpleasant behaviors displayed by a person, and not necessarily steam from stigma to a given diagnosis. Assessments for this study were performed in a mental health institution by medical personnel; this could have influenced participant’s responses regarding certain sources of stigma. Specifically, stigma towards mental health professionals could be undervalued. It should be highlighted that stigma surrounding BPD in Mexico may be shaped by societal attitudes, traditional values, and mental health perceptions [45]. Mental health issues have historically been viewed with suspicion or shame, particularly due the limited mental health education in Mexico. This lack of understanding often leads to BPD being perceived as a character flaw rather than a legitimate disorder, contributing to its stigmatization and unfair labeling and women affected by the disorder may internalize the guilts and shame associated with popular beliefs of individuals as manipulative, attention-seeking, or unstable. Thus, it is suggested that future studies include an analysis of the sociocultural environment in which patients live to understand its influence on internalized stigma.

## Conclusions

Internalized stigma is relevant in terms of the severity and prognosis of BPD. Its structure seems to be affected by the source where stigma comes from. Further investigations on this topic could help identify how self-stigma is constructed and whether interventions in the support network could help preventing it.

## Availability of Data and Materials

The data presented in the present manuscript is available on request from the corresponding author.
